# Uncommon cause for anterior knee pain - Aggressive aneurysmal bone cyst of the patella

**DOI:** 10.1186/1758-2555-2-9

**Published:** 2010-04-01

**Authors:** Maurice Balke, Nicolas Dedy, Jonas Mueller-Huebenthal, Dennis Liem, Jendrik Hardes, Juergen Hoeher

**Affiliations:** 1 Department of Trauma and Orthopedic Surgery, University of Witten-Herdecke, Cologne-Merheim Medical Center, Ostmerheimer Str. 200, 51109 Cologne, Germany; 2 Department of Orthopaedic Surgery, University Hospital Muenster, Albert-Schweitzer-Str. 33, 48149 Muenster, Germany; 3 Department of Radiology, Cologne Triangle, Ottoplatz 1, 50679 Cologne, Germany; 4 Division of Sports Medicine, Trauma Department, Hospital Cologne-Merheim, Ostmerheimer Str. 200, 51109 Cologne, Germany

## Abstract

A 56-year-old man presented with a two month history of increasing anterior knee pain without previous trauma. As usual we recommended physiotherapy with stretching exercises of the quadriceps muscle. Since symptoms did not improve after 6 weeks MRI was performed. Surprisingly a hyperintense lobulated mass of the patella with small fluid-filled cavities at the inferior pole was revealed. We performed an open biopsy to exclude any malignancy and diagnosed an aneurysmal bone cyst. Further examination with CT scans showed an aggressive behaviour with cortical breakthrough.

We performed an intralesional curettage with additional high-speed burring and bone cement packing. Sixteen months later the patient was free from any complaints and without signs of local recurrence.

Primary bone tumors of the patella are extremely rare and occurrence of aneurysmal bone cysts in this localization is very uncommon. This case report indicates that although anterior knee pain is a very frequent and usually harmless symptom, it is essential to consider that it might also be caused by more severe disorders such as bone tumors.

## Background

Anterior knee pain usually is an insistent but harmless symptom [1]. Rarely the pain is caused by severe conditions such as bone tumors [2-4]. The majority of bone tumors affecting the patella are benign lesions with giant cell tumor of bone and chondroblastoma being the most common [2,4,5]. The occurrence of aneurysmal bone cysts (ABCs) in the patella is anecdotal [6-11]. Most published cases describe "latent" (stage I) or "active" lesions (stage II) according to the staging system of benign skeletal tumors by Enneking [12]. Aggressive (stage III) lesions as presented here occur even more rarely [11].

The subject of this report gave informed consent that the data of this case would be submitted for publication.

## Case report

**Figure 1:**
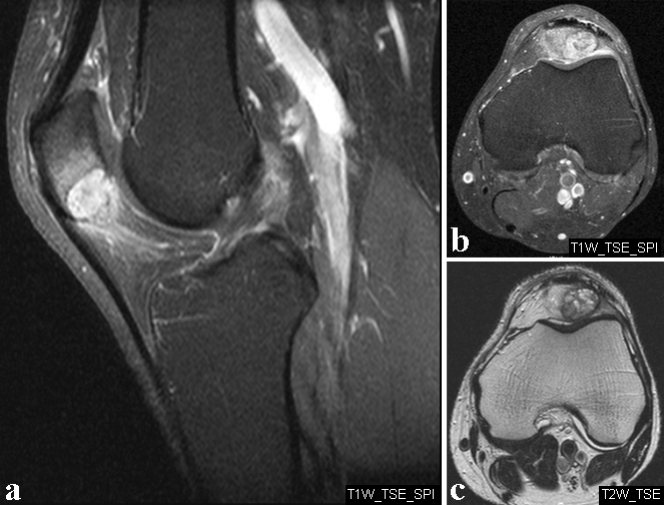
**Magnetic resonance imaging of the left knee**. MRI of the left knee showing a hyperintense lesion within the inferior pole of the patella. (**a**) sagittal T1, (**b**) axial T1, and (**c**) axial T2 weighted sequences.

**Figure 2:**
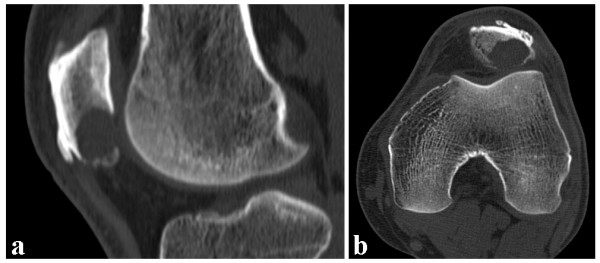
**Computed tomography of the left knee**. CT scan of the left patella showing an osteolytic lesion within the inferior pole of the patella. Note the cortical thinning/breakthrough as a sign for the aggressiveness of the lesion. (**a**) sagittal, (**b**) axial sequence.

**Figure 3:**
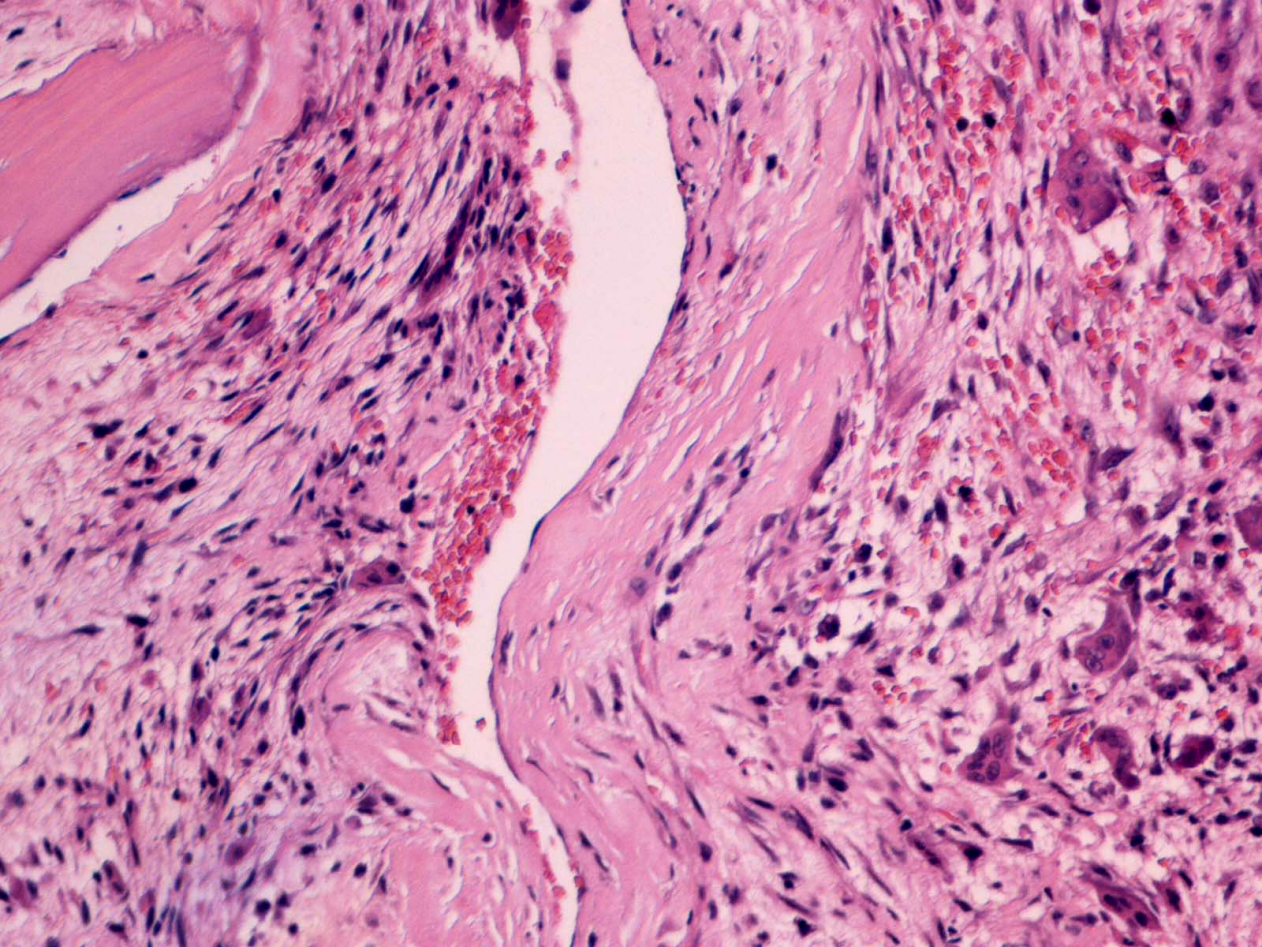
**Histology sample**. Histology sample showing a typical picture of aneurysmal bone cyst. Hematoxyline-Eosine stain, 20×. Cavernomatous spaces lined with endothelial cells and containing extravasated red blood cells. Note the multinucleated giant cells.

In May 2008 a 56-year-old man presented at our outpatient clinic with a two month history of increasing anterior knee pain of the left leg without previous trauma. The patient did not perform any sports on a regular basis. Physical examination revealed a full active and passive range of motion of the left knee, but maximal flexion was painful. There was no effusion or swelling but moderate tenderness on palpation of the inferior pole of the patella. Physiotherapy with stretching exercises of the quadriceps muscle did not improve symptoms. Magnetic resonance imaging (MRI) was performed and showed a hyperintense lobulated mass with small fluid-filled cavities at the inferior pole of the patella (Figure 1). An additional CT scan revealed an osteolytic lesion with endosteal scalloping and cortical thinning (Figure 2). In June 2008 the patient was referred to a musculoskeletal tumor centre where a biopsy according to the guidelines of the musculoskeletal tumor society [13] was performed. Histology showed bony fragments with fibrohistiocytic proliferations, loosely arranged spindle cells, and several multinuclear giant cells. The cavernomatous spaces were lined with endothelial cells and contained red blood cells (Figure 3). Although the typical fluid-fluid levels on the MRI were not visible the tumor was diagnosed as a benign primary aneurysmal bone cyst (ABC). Curettage with burring and bone grafting was recommended but the patient deferred the surgery to September 2008 for occupational reasons.

**Figure 4:**
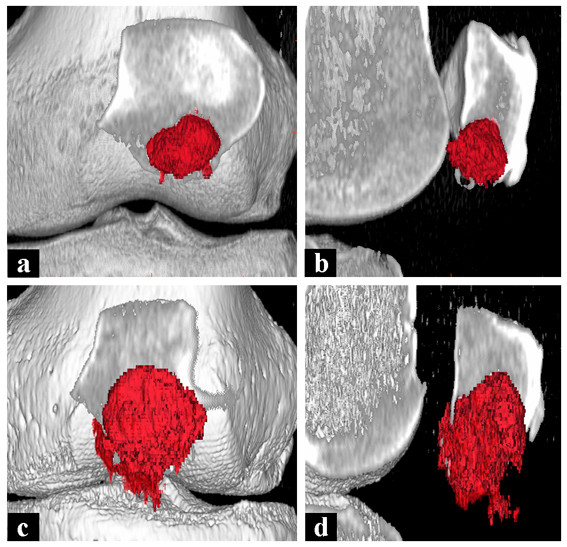
**CT-scans with three-dimensional reconstruction and volume analysis**. Three-dimensional reconstructions of CT-scans showing the significant progress of the lesion from May (**a **+ **b**) to August 2008 (**b **+ **c**).

Because of the long interval we performed another CT scan of the patella that revealed a significant progression of growth from initially 2.5 ml (Figure 4a + b) to approximately 9 ml (Figure 4c + d) with cortical breakthrough. Surgery revealed a lobulated cavity filled with a mixture of blood and solid red tissue as well as a fracture of the cortex at the inferior patellar pole, with the covering cartilage still intact. We performed intralesional curettage through a lateral approach with additional high-speed burring and lavage with hydrogen peroxide. Due to the aggressiveness of the lesion we decided to fill the cavity with bone cement instead of bone graft to reduce the likelihood of recurrence. Histology confirmed the initial diagnosis of benign ABC. The further treatment consisted of full weight-bearing of the left leg and physiotherapy to regain full range of motion.

**Figure 5:**
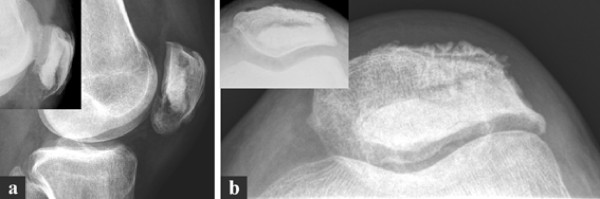
**Radiographs of the left patella after bone cement packing**. Radiographs of the patella 17 months after surgery. The small X-rays were performed 1 week after filling of the cavity with bone cement. Note the lack of the inferior cortex of the patella (**a - small**), which is remodelled at last follow-up. **b **shows degenerative changes at last follow-up but without clinical symptoms.

At last follow up in February 2010 the patient was free of complaints and had a full range of motion. The standard radiographs showed regular placement of the bone cement without evidence of local recurrence (Figure 5). After 17 months the inferior cortex is remodelled (Figure 5a), but there are also degenerative changes in the femoropatellar joint (Figure 5b). However this did not cause clinical symptoms. The knee society score, the Lysholm score and the WOMAC osteoarthritis index showed excellent results with 100 points each.

## Discussion

Anterior knee pain is a common clinical symptom. It can be caused by a variety of anatomical structures such as patellofemoral cartilage, anterior capsule, subchondral bone, synovial tissue, infrapatellar fat pad, and patellar tendon [1]. The pain may be related to several disorders such as patellar tendonitis [14], hypertrophic synovial plicae or so called chondromalacia patellae [15]. In most cases no specific cause is identified.

However, anterior knee pain may also be the first symptom of a bone tumor. Primary intraosseus lesions of the patella are very rare and a number of differential diagnoses need to be considered [2-4]. The majority of these lesions are benign with giant cell tumor of bone and chondroblastoma being the most common diagnoses [2,4,5].

Aneurysmal bone cysts account for 1% to 2% of all primary bone tumors, usually present in the first two decades of life, and exhibit a slight female preponderance [16]. ABCs are usually osteolytic lesions consisting of a lobulated blood-filled cavity including giant cell-rich soft tissue. On standard radiographs they present as an osteolytic lesion. Magnetic resonance imaging typically shows fluid-fluid levels, ABCs usually occur within the metaphyses of the long bones; affection of the patella is seldom [6-11].

According to Enneking benign bone tumors can be graded in three stages: stage I lesions are latent with a well defined border, stage II lesions are active with expanding growth causing cortical thinning, and stage III lesions are aggressive with cortical breakthrough and invade the soft-tissue occasionally [12]. Most of the reported ABCs of the patella were stage I or II lesions [6-9,11]. Due to cortical breakthrough our case is referred to as a stage III lesion. The generally recommended treatment consists of curettage and autogenous bone grafting for stage I and II lesions, sometimes added by chemical adjuvants [16,17]. Some authors recommend wide resection/total patellectomy for stage III lesions [6].

Since the typical fluid-fluid levels were not visible in the MRI, we performed an open biopsy to exclude any malignancy. Finally, we treated our patient with curettage, additional burring with a high-speed burr and lavage of hydrogen peroxide. Due to the aggressiveness of the lesion we decided to used bone cement packing as an additional adjunct. This combination has been shown to significantly reduce the local recurrence rate in biologically comparable tumors such as giant cell tumors of bone [5]. Despite the cortical breakthrough total patellectomy or resection of the inferior patellar pole seemed to be an over treatment, especially because the main retropatellar surface was not affected. At last follow-up one year after surgery the patient was free from complaints and without any signs of recurrence.

In conclusion, although anterior knee pain is a usually harmless symptom, it is essential to consider that it might also be caused by more severe disorders such as bone tumors.

## Consent

Written informed consent was obtained from the patient for publication of this case report.

## Competing interests

The authors declare that they have no competing interests.

## Authors' contributions

All authors co-wrote the paper and discussed the results and commented on the manuscript. All authors read and approved the final manuscript.
